# *Candida albicans* Hyphae: From Growth Initiation to Invasion

**DOI:** 10.3390/jof4010010

**Published:** 2018-01-11

**Authors:** Jigar V. Desai

**Affiliations:** Fungal Pathogenesis Section, Laboratory of Clinical Immunology and Microbiology, National Institute of Allergy and Infectious Diseases (NIAID), National Institutes of Health (NIH), Bethesda, MD 20892, USA; jigarkumar.desai@nih.gov; Tel.: +1-301-761-6585

**Keywords:** *Candida albicans*, hyphae, invasion

## Abstract

*Candida albicans* is a commensal resident of the human gastrointestinal and genital tracts. Under conditions such as dysbiosis, host immune perturbances, or the presence of catheters/implanted medical devices, the fungus may cause debilitating mucosal or fatal systemic infections. The ability of *C. albicans* to grow as long filamentous hyphae is critical for its pathogenic potential as it allows the fungus to invade the underlying substratum. In this brief review, I will outline the current understanding regarding the mechanistic regulation of hyphal growth and invasion in *C. albicans*.

## 1. Introduction

The fungal hypha is a manifestation of anisotropic growth: a non-uniform cellular volume expansion along a polarized axis. In higher eukaryotes, the cell-types arising in this manner serve diverse roles in organismic life-style. For example, in plants, the sclerenchymic fibers provide structural support, while root hairs developed from the trichoblasts are critical for water absorption [1]. In animals, anisotropic expansion regulates the precise patterning in a developing tissue [2]. In fungi, anisotropic growth as hyphae, a trait proposed to be evolved from the flagellar protists upon terrestrial colonization, not only compensates for the motility loss but also functions in nutrient acquisition, niche colonization and mating [3,4]. In the human pathogenic fungus *Candida albicans*, hyphal growth is clinically relevant as it is a critical driver of pathogenesis in symptomatic mucosal infections such as oral thrush and vaginal candidiasis, as well as fatal systemic infections. In these infections, the infected tissues often contain invading hyphae and in agreement, the *C. albicans* hyphal-defective mutants show defects in their virulence [5,6,7,8]. Given the importance of hyphae in virulence, hyphal morphogenesis in *C. albicans* has been an area of rigorous investigation.

Fundamentally, hyphal growth requires symmetry breaking and establishment of a polarity axis along which anisotropic cellular expansion can occur. Upon establishment of the axis for anisotropic expansion, hyphal growth initiates with emergence of a germ-tube from the area marked by polarity landmarks [9,10,11,12,13]. The germ-tube then extends and grows exclusively via growth at the tip. The exclusive tip expansion requires a plastic cell-wall at the tip, with elastic cell-walls laterally. The hydrostatic-pressure driven cytoplasmic forces expand the plastic cell-wall at the tip and thus drive the hyphal growth. In agreement, *C. albicans* hyphal growth rate is substantially retarded in hypertonic media [14]. The cytoskeleton, on the other hand, maintains directional growth via directing the vesicular flow for tip expansion, without much contribution to the cell-wall expansion forces. Indeed, actin disruption in growing hyphae, although disrupts the hyphal growth, still leads to isotropic cell-wall expansion and hyphal tip swelling [15]. Moreover, concurrent to the tip-expansion, the *C. albicans* hyphae undergoes mitotic cell division without constriction at the mother- and daughter cell boundaries and cell separation, thus leading to parallel-walled hyphal formation [8,16]. Hyphal growth also leads to a hyphal-specific transcriptional program which paves the way towards upregulation of virulence-related processes, such as increased adherence, biofilm formation and cellular invasion [17,18]. In the sections below I will highlight the key findings which have provided invaluable insights into the molecular regulation of the hyphal growth, directionality maintenance and invasion.

## 2. Hyphal Initiation, Elongation and Directionality Maintenance

**Figure 1:**
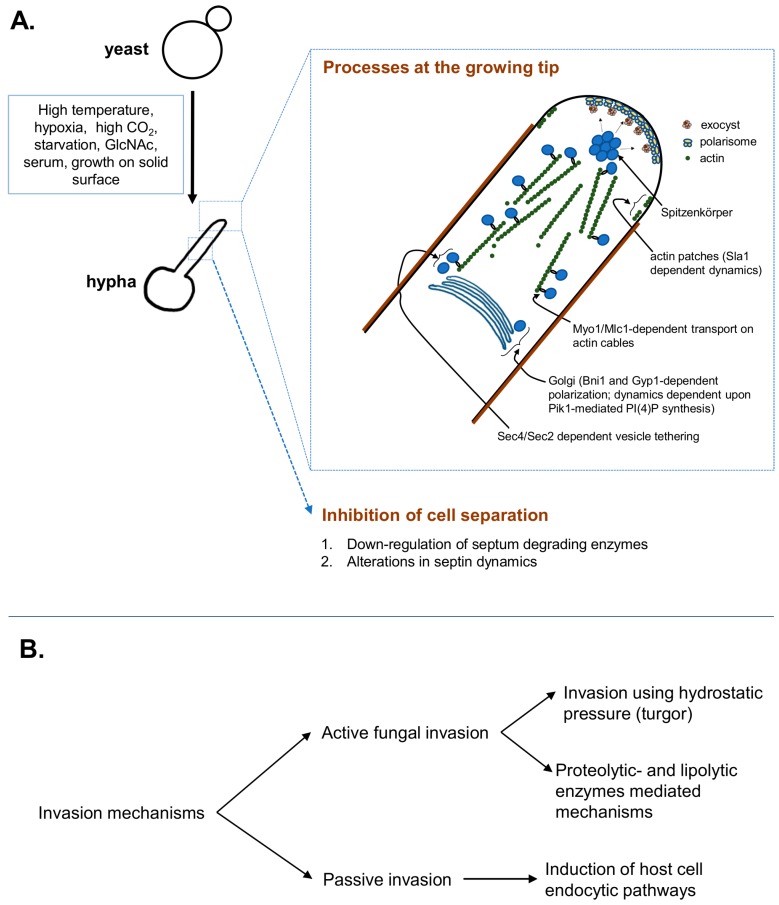
Schematic representation of *Candida albicans* hyphal growth and depiction of the major invasion mechanisms. (**A**) Distinct environmental cues signal hyphal initiation, where coordinated processes of Golgi polarization, cytoskeletal rearrangements, polarized exocytosis at the plastic tip, endocytosis and cell separation inhibition are responsible for efficient morphogenesis; (**B**) The *C. albicans* hyphae invade the substratum actively, using turgor-driven force/enzymatic substratum digestion or inducing its own endocytosis by the host cells. Detailed description of the key players and their regulatory control is described under the text.

At the growing hyphal tip, a steady delivery of exocytic vesicles arriving on the actin cables maintains the supply for cell-wall expansion. As shown in Figure 1, the actin cables, organized along the growth axis, maintain the delivery of exocytic vesicles emerging from the Golgi, which is polarized towards hyphal-tip in the formin (Bni1)-dependent manner [19]. Golgi also depends on Phosphatidylinositol 4-phosphate (PI(4)P), synthesized by phosphatidylinositol kinase (Pik1), to maintain vesicular trafficking and polarized hyphal growth via regulation of its own dynamics [20]. The vesicles, emerging from the Golgi, are tethered to the actin cytoskeletal cables via a Rab GTPase- Sec4 in the guanine nucleotide exchange factor (GEF)- Sec2 dependent manner, which are transported along the actin cables via a myosin motor and its regulatory subunit complex- Myo1/Mlc1 [15]. These dynamic vesicles, marked by the fluorescent-protein-tagged-Sec4 are observed at an apical spot called Spitzenkörper [15]. Spitzenkörper not only marks the highly dynamic exocytic- but also endocytic vesicles, traversing to and from the cell-membrane [21]. The exocytic vesicles then tether to the multiprotein exocyst complex, which then allows vesicles to dock at the plasma membrane for delivery of the cargo [22]. These Spitzenkörper-traversing exocytic vesicles provide the membrane and cell-wall synthesis enzymes at the growing hyphal tip [23]. The vesicles-supplied cell-wall synthesizing enzymes extrude the cell-wall building blocks from cytoplasm at the growing tip [23], while the actin patches regulate endocytosis of possibly excess) membrane and the cell-wall synthesis enzymes from the lateral sides, to avoid isotropic growth [23]. At the hyphal tip, the protein complex called polarisome is also essential for anisotropic growth via its roles in organization of actin nucleation site. Polarisome marks an apical area at the hyphal tip that displays stable localization of the scaffolding protein Spa2, a formin-actin binding protein Bud6, and the Rho GTPase Cdc42 [15,21].

The small GTPases of the Ras- and Rho- family play essential roles in hyphal extension [24,25]. Their activity is dependent on efficient guanosine triphosphate/guanosine diphosphate (GDP/GTP) recycling; regulated via the guanine exchange factors (GEFs), GTPase activating proteins (GAPs) and guanine nucleotide dissociation inhibitors (GDIs). The Rho GTPase Cdc42 is a master regulator of polarized hyphal growth in *C. albicans* and is required for efficient hyphal formation [26]. Cd42 activity is focused at the apex of the hyphal tip by the Ras-like GTPase- Rsr1 which is also required for invasive hyphal growth [11,27,28]. Cdc42 has three GAPs (Rga1, Rga2, Bem3), a GEF (Cdc24) and a GDI (Rdi2) that act to control Cdc42 activity and thus hyphal growth [29]. As in the budding yeast, upon activation by Cdc24, Cdc42 through the scaffolding protein- Bem1, regulates the formin activity for polarized growth [30]. This Cdc42-dependent polarity establishment in *C. albicans* is Ca^2+^ dependent, through Ca^2+^ binding region of Cdc24 [29]. In agreement, the Ca^2+^ channels (Cch1 and Mid1) are required in tropic behavior by *C. albicans* hyphae [31]. Similar to Cdc42, the GTPase Rsr1’s activity is maintained via the GEF (Bud5) and the GAP (Bud2). Bud5 localization at the apex, while subapical localization of Bud2, focuses Rsr1 activity at the hyphal tip [27], where it directly interacts with the exocyst component Sec15 for polarized exocyst localization [32]. On a solid surface, the surface attached, tip-down growth as well hyphal reorientation upon obstacle encountering is also regulated via Rsr1/Cdc42 [29,33]. Another Rho GTPase- Rac1, on the other hand is required for invasive filamentous growth in a manner dependent upon the GEF Dck1 and an Engulfment and celL MOtility (ELMO) homologue Lmo1 [34,35].

The hyphal extension machinery including the small GTPases are under tight regulation by the cell-cycle associated cyclins and cyclin-dependent kinase 1 (Cdk1/Cdc28). In *C. albicans*, the three G1 cyclins (Ccn1, Cln3 and Hgc1) and two G2 cyclins (Clb2 and Clb4), have essential roles in morphogenesis [36]. These cyclins cooperate with each other and other protein kinases to fine-tune the processes for efficient tip expansion and inhibition of cell separation. For example, the polarisome is maintained at the tip by cooperative actions of the Clb2- and Hgc1/Cdc28 [37]. Moreover, Hgc1/Cdc28 also phosphorylates the Cdc42 GAP-Rga2, prevents its tip-localization and ensures Cdc42 activity at the tip for efficient hyphal growth [38]. Cln3/Cdc28 phosphorylates an endocytosis protein Sla1 to regulate actin patch dynamics; rapid actin patch dynamics are observed in growing hyphae [39]. Direct phosphorylation of Exo84 and Sec2 by Hgc1/Cdc28 controls exocyst assembly and Golgi-emerging vesicle tethering to the cytoskeleton, respectively [22,40]. In addition, an NDR (nuclear Dbf-related kinase) Cbk1 via interaction with its activator protein Mob2 (activated in Cdc28-dependent manner) also regulates polarisome maintenance at the tip [41]. For cell separation inhibition subsequent to cytokinesis, precise regulation of septum formation/degradation is critical. The GTP-binding proteins called septins are critical for their roles in septum formation, cytokinesis and polarized growth [42]. *C. albicans* contains five different septins: Cdc3, Cdc10, Cdc11, Cdc12 and Sep7, with critical roles in hyphal morphogenesis [42,43,44,45]. The septins first localize at tip of the growing germ-tube, which emerges from the site marked by landmark proteins such as Int1 and Rsr1 [9,16,46]. The septins remain there until nuclear division, after which a detectable signal appears in form of a ring [16]. In growing hyphae, the septin rings display stably localized Cdc3-Cdc12-Sep7 with a dynamic exchange of Cdc10 [44]. This Sep7-dependent frozen-septin core is necessary for inhibiting the phosphatase Cdc14-mediated functions in cell separation [44]. As the nuclear division progresses further along the mitotic trajectory, the divided nuclei translocate into the daughter- and mother cell compartments, concurrent to which the septin ring divides into two [16]. The divided septin rings pave the way towards formation of the chitinous septum, which is kept intact via downregulation of septum degrading enzymes [8]. In hyphae, the septum-degrading enzymes (SDEs) are down-regulated via Hgc1/Cdc28-dependent phosphorylation of the transcription factor Efg1 that restricts the transcription factor Ace2 from binding to the SDE-promoters [47]. On the other hand, Ace2, in transcription-independent manner, associates with Cbk1 at the septin ring and regulates Sep7 incorporation into the ring and thus the ring stability [48]. Efg1, in addition to its roles in cell separation inhibition, is the master regulator of hyphal morphogenesis that directly regulates expression of major hyphal regulators and hyphal associated genes [49,50,51]. Many of the environmental cues that signal for hyphal initiation act through Efg1 to modulate hyphal specific gene expression [7]. Thus, under hyphal stimulating environmental conditions, the precisely regulated events of cell-wall extension via directed vesicle delivery and inhibition of cell separation drives hyphal growth.

Among the environmental cues that are known to signal hyphal initiation (and maintenance) in *C. albicans*, starvation, increased pH, hypoxia, high CO_2_, GlcNAc, serum and growth on solid surface are the most important. The molecular details regarding how these environmental signals are integrated to affect the hyphal growth are now emerging. Among the different environmental cues; CO_2_, serum and high temperature activate an adenylate cyclase Cyr1, which leads to cAMP generation and activation of protein kinase A (PKA) complex [7]. In case of temperature-mediated morphogenesis, heat-shock protein 90 (Hsp90), at a higher temperature, relieves the otherwise repressed PKA [52]. On the other hand, CO_2_ upon conversion into HCO_3_^2−^, directly binds Cyr1, increases cAMP production and activates PKA [53]. Consequently, the activated PKA relieves the hyphal transcriptional repression via down-regulating of transcription factor Nrg1 [54]. Additionally, other mechanisms, exemplified by Cup9/Ubr1/Sok1 and Cbk1/Ssd1 mediated Nrg1 repression, acts in parallel to allow hyphal initiation [55,56]. The Cup9/Ubr1/Sok1 pathway may also function in Nrg1 repression in filamentation on solid media [17]. Among the PKA-independent pathways, the alkaline pH mediated morphogenesis is regulated via the transcription factor Rim101 [57], while morphogenesis on solid surface is regulated via Cek1 in Rac1/Dck1-dependent manner [34,35,58]. In addition to the transcriptional impact, activated PKA pathway also phosphorylates Gyp1 (a Rab GTPase activating protein) for an enhanced association to Myo2 for Golgi polarization towards the growing hyphal tip [59]. As the hypha is elongating, discrete chromatin alterations maintain the hyphal extension, despite normalized Nrg1 levels. It is achieved via the GATA transcription factor- Brg1 mediated recruitment of Hda1, which restricts Nrg1 access to the promoters of hyphal genes [60]. Starvation sensing via the Target of Rapamycin kinase- Tor1 also acts in obstructing Nrg1 promoter binding via Brg1 [61]. In addition to the environmental cues highlighted above, cell cycle duress or stress due to depletion of essential genes’ encoded proteins also induce hyphal morphogenesis which bypass many of the hyphal transcriptional activators [62,63]. For a detailed outline of signaling and transcriptional regulation of hyphal morphogenesis, the readers are directed to the excellent reviews elsewhere [7,8,54].

## 3. Hyphal Invasion

As highlighted under introductory paragraphs, the cytoplasmic forces driven by hydrostatic pressure (turgor), similar to the other fungi [64], may drive *C. albicans* hyphal invasion as well. In addition, *C. albicans* also harbors proteases that may directly assist in invasion [5,65]. In case of the hydrostatic pressure or turgor, as it is isotropic in nature, the force is exerted uniformly across all direction. In a growing hypha, actin cytoskeletal disruption leads to isotropic hyphal tip swelling, which clearly demonstrates the isotropic nature of turgor [15]. Which molecule contributes to turgor? The plant pathogenic fungi utilize glycerol for invasive growth [66], however *C. albicans* mutants which are defective in accumulating glycerol are not defective in hyphal growth [67]. The glycerol defective hyphae, on the other hand, are defective in invasion in vitro, as well as in virulence in a mouse model of intraabdominal candidiasis [68,69], implying a putative role for glycerol in supplementing additional forces needed to invade. Since glycerol has no roles in hyphal morphogenesis, additional osmolytes that serve the critical functions in hyphal morphogenesis, as well as invasion, still remain to be defined. Regardless, mathematical modeling suggests that if hyphal turgor (due to overall cytoplasmic content) reaches a magnitude that is equivalent to or greater than the elastic modulus of the substratum, the hyphae can initiate invasion; subsequent to which, the invasion rate depends upon turgor, substrate’s elastic modulus and cell-wall extensibility at the tip [68]. Direct real-time imaging of hyphal cells pushing against microfabricated obstacles revealed invasion forces resulting from turgor of ~1.2 MPa [33]. Interestingly, the real-time imaging experiments also showed that hypha grows along the substrate, tip-down with Spitzenkörper oriented towards the substrate, without invasion [33]. Upon encountering the substrate perpendicular to its growth axis, the hypha pushes into the material and then reorients the growth trajectory along the surface, in Cdc42 and Rsr1 dependent manner [29,33]. It is yet uncharacterized at what point, then, invasion occurs and what physicochemical cues regulate surface-associated versus invasive growth switch. Based on the data so far, it can be postulated that at-first, the three-dimensional surface associated growth as a biofilm ensues which then signals the hyphae in contact with the substrate to invade.

In addition to the fungal mediated active invasion mechanisms, the host tissue invasion elicits a host-assisted invasion mechanism. For example, the endothelial and epithelial cells actively endocytose *C. albicans* via *N*-cadherin, *E*-cadherin, and EGFR/HER2 [70,71]. The endocytic uptake is mediated via fungal cell wall proteins such as Als3/Ssa1 which, upon binding to aforementioned host proteins, induce endocytic uptake in clathrin and endothelial Septin-7 dependent manner [70,71,72,73]. The host cytokines can also affect the endocytic invasion. For example, Solis et al. recently showed that IFN-γ leads to production of kynurenins, which act on the aryl hydrocarbon receptor (AhR) for a prolonged activation and inhibits EGFR-dependent fungal endocytosis [74]. Future studies aimed at understanding how the other host immune mediators such as cytokines, chemokines affect epithelial endocytic fungal invasion will provide further insights into this mechanism. The tissue-resident mononuclear phagocytes also rapidly take up the fungus via phagocytosis [75,76,77]. Ex vivo it has been shown that the hyphae can escape the phagocytes [78,79], however how much this contributes to subsequent tissue invasion is not known.

In order to understand the relative contributions of each invasion mechanisms in cellular invasion, experiments have been carried out using dead fungus and/or host-cell cytoskeletal disruption, which showed differential utilization of invasion mechanisms depending upon the cell-type in question [80,81]. For example, enterocyte invasion required major contribution from active mechanism while oral epithelial cell invasion utilized endocytic uptake as well [81]. Among the different genes that mediate the invasion, the glycerol-deficient mutants are defective in damaging oral epithelial cells and are defective in invading enterocytes ex vivo [69,82]. Similarly, other genes such as *RSR1* (Ras-like GTPase), *BUD2* (Rsr1 GAP), *CKA2* (protein kinase acting in the calcineurin pathway), *BCR1* (transcription factor responsible for adhesin and biofilm-related gene expression) and *HWP1* (hyphal wall protein and adhesin) are also critically required for invading the enterocytes ex vivo [69]. The above genes mediate enterocytes invasion but are dispensable for oral epithelial invasion. On the other hand, genes in the cAMP/PKA pathway, *RAS1* (Ras-family GTPase), *RIM101* (alkaline pH induced hyphal regulator), and *HGC1* (G2 cyclin) are required for invasion into the both cell-types [69]. The relative contribution (and cooperation) of lytic vs. turgid and passive endocytic mechanism in tissue invasion remains to be characterized.

In summary, *C. albicans* employs a combination of strategies to invade the underlying substrata, including abiotic substrata of implanted medical devices. In host tissue invasion, cooperation between host-assisted uptake and the active fungal mediated invasion is likely. Experiments using cell monolayers showed the active invasion mechanism to be a major contributor [80], however the relative contributions of each, in vivo, are yet to be examined. Further studies will illuminate the molecular regulation of invasion mechanisms and their impacts on pathogenicity.
